# Performance Evaluation of a Multichannel All-In-One Phantom Dosimeter for Dose Measurement of Diagnostic X-ray Beam

**DOI:** 10.3390/s151128490

**Published:** 2015-11-11

**Authors:** Hyesu Jeon, Wook Jae Yoo, Sang Hun Shin, Guwon Kwon, Mingeon Kim, Hye Jin Kim, Young Beom Song, Kyoung Won Jang, Won Sik Youn, Bongsoo Lee

**Affiliations:** 1 School of Biomedical Engineering, College of Biomedical & Health Science, BK21 Plus Research Institute of Biomedical Engineering, Konkuk University, 268 Chungwon-daero, Chungju-si, Chungcheongbuk-do, 380-701, Korea; E-Mails: jeonhyesu8709@gmail.com (H.J.); wonzip@kku.ac.kr (W.J.Y.); shshin9431@gmail.com (S.H.S.); lucky7568@naver.com (G.K.); ganzerkim@gmail.com (M.K.); hye_jin0723@naver.com (H.J.K.); dudqja820@gmail.com (Y.B.S.); kko988@hotmail.com (K.W.J.); 2 Research & Development Center, JPI Healthcare, Osongsaengmyeong 1-ro, Osong-eup, Cheongwon-gun, Chungcheongbuk-do, 363-951, Korea; E-Mail: younws@JPI.co.kr

**Keywords:** dosimeter, dose distribution, radiation diagnosis, scintillation signal, chest phantom

## Abstract

We developed a multichannel all-in-one phantom dosimeter system composed of nine sensing probes, a chest phantom, an image intensifier, and a complementary metal-oxide semiconductor (CMOS) image sensor to measure the dose distribution of an X-ray beam used in radiation diagnosis. Nine sensing probes of the phantom dosimeter were fabricated identically by connecting a plastic scintillating fiber (PSF) to a plastic optical fiber (POF). To measure the planar dose distribution on a chest phantom according to exposure parameters used in clinical practice, we divided the top of the chest phantom into nine equal parts virtually and then installed the nine sensing probes at each center of the nine equal parts on the top of the chest phantom as measuring points. Each scintillation signal generated in the nine sensing probes was transmitted through the POFs and then intensified by the image intensifier because the scintillation signal normally has a very low light intensity. Real-time scintillation images (RSIs) containing the intensified scintillation signals were taken by the CMOS image sensor with a single lens optical system and displayed through a software program. Under variation of the exposure parameters, we measured RSIs containing dose information using the multichannel all-in-one phantom dosimeter and compared the results with the absorbed doses obtained by using a semiconductor dosimeter (SCD). From the experimental results of this study, the light intensities of nine regions of interest (ROI) in the RSI measured by the phantom dosimeter were similar to the dose distribution obtained using the SCD. In conclusion, we demonstrated that the planar dose distribution including the entrance surface dose (ESD) can be easily measured by using the proposed phantom dosimeter system.

## 1. Introduction

As a radiation diagnostic procedure, posteroanterior (PA) chest radiography is the most commonly applied methodology and it is increasingly important in implications for delivering excessive radiation to patients to provide diagnostically acceptable images. In practice, it has been noticed that radiological technologists have tended to prefer high exposure in X-ray exams because it provides high-quality images. Although it is known that the individual patient dose in PA chest radiography is comparatively low, it can significantly affect the cumulative dose with the frequent use of radiography [1]. While patients are exposed to excessive radiation, their exposure dose has been highlighted globally. Furthermore, apprehension about possible harm to people caused by radiation exposure has risen as the clinical use of diagnostic procedures increases. The radiological technologists have been demanding dose information [2,3]. 

The entrance surface dose (ESD reported in mGy) value is specified as a useful descriptor to evaluate the absorbed dose of the patient in diagnostic radiology and it is generally defined as the absorbed dose detected at the point of intersection of the central X-ray beam axis with the patient or phantom surface, including backscattered radiation [2,3]. In practice, however, the absorbed dose is delivered unevenly in a beam irradiation field because the intensity distribution in an X-ray beam is not uniform due to the influence of the heel effect. Hence, this may lead to the possibility that the ESD value measured at the center of the beam irradiation field can be underestimated because the higher absorbed doses are practically delivered at the other points. It means that an excessive radiation dose can be delivered to a part of the patient's body even though the measured ESD value is under the diagnostic reference level (DRL). 

In recent years, a multitude of studies have been conducted to measure the radiation dose in order to optimize radiographic procedures to achieve as reasonably low a radiation dose as possible, and the results have indicated possibilities to reduce the dose without detriment to image quality [4]. For successful and efficient medical effects, safety and quality are imperative. In addition, precision and sensitivity of the operating characteristics of the imaging system are related to benefits for patient health [5]. In diagnostic radiology, the correlation of image quality with patient absorbed dose must be set to provide acceptable image quality at the lowest practicable dose level; this balance is usually defined by the exposure parameters of the X-ray imaging system. The periodic assessment of exposures in the comprehensive quality assurance (QA) of an X-ray imaging system is an important part to identify problems and to ensure that diagnostic quality is maintained without over-exposure [6].

For *in situ* and real-time measurements of the absorbed dose at the measuring points, various kinds of clinical dosimeters have been developed, including ionization chambers (ICs) and semiconductor dosimeters (SCDs). However, in the cases of ICs and SCDs, they have a large sensing volume and their sensing probes are composed of high-atomic-number materials. Accordingly, most conventional ICs and SCDs can generate distracting image artifacts and interfere with the examination of X-ray imaging [7]. To overcome the problems of existing dosimeters, various scintillating fiber-optic dosimeters (SFODs) based on plastic scintillating fibers (PSFs) and plastic optical fibers (POFs) have been investigated as promising candidates because SFODs have many useful dosimetric qualities, such as a small sensing probe with low-atomic-number materials (*i.e.*, near tissue-equivalent materials), a light weight, adequate flexibility, immunity to electromagnetic interference (EMI), remote operation capabilities, and real-time measurement [3,7,8,9]. Several studies on the performance evaluation of a fiber-optic-coupled dosimeter (FOC) for use in radiation diagnosis have been reported by Hintenlang *et al.* [9,10,11]. However, the design of the phantom dosimeter proposed in the present study is significantly different from that of the existing FOC system fabricated by Hintenlang *et al.* Recently, various experimental studies on the real-time measurement of ESD using a SFOD during radiation diagnosis have been carried out by our research team [3,7,8]. In previous experiments, we developed a SFOD with two sensing probes to infer the ESD value at the center of a beam irradiation field without the impediment of X-ray imaging [8] and demonstrated that a SFOD can be used for the measurement of real-time ESD [7]. However, it is difficult to rapidly measure the two-dimensional (2D) dose distribution of the heel effect affecting the X-ray beam used in radiation diagnosis with existing SFOD systems. 

In the present study, to measure the planar dose distribution and to apply it to the QA of a diagnostic X-ray imaging system, a multichannel all-in-one phantom dosimeter system was developed using nine sensing probes, a chest phantom, an image intensifier, and a complementary metal-oxide semiconductor (CMOS) image sensor. If the beam quality of the X-ray exceeds the standard values, the dose measurement may be invalid and the patient can be exposed to an unnecessary dose [12]. Accordingly, the phantom dosimeter system was exploited to measure the beam profile of the X-ray used in radiation diagnosis to acquire information on the beam quality. Using a beam profile, we obtained the dose distribution that can be applied to check the X-ray beam quality. Furthermore, we selected a suitable measuring point according to the relationship between each sensing probe for calculating the ESD values. 

## 2. Materials and Instruments

### 2.1. Fabrication of Nine Sensing Probes

A PSF (BCF-12, Saint-Gobain Ceramic & Plastics, Hiram, OH, USA), which has a core/single-clad structure with a diameter of 1 mm, was employed to generate scintillation signals as a sensitive material of the sensing probes. The core is a synthetic material of polystyrene and fluorescent dopants and the cladding material is polymethylmethacrylate (PMMA). The refractive index of the core is 1.60 and the numerical aperture (NA) is approximately 0.58. The decay time and the emission peak are 2.7 ns and 435 nm, respectively. To transmit the scintillation signals generated from the PSF in the sensing probe, a POF (GH 4001, Mitsubishi Rayon, Tokyo, Japan) with a step-index and multimode characteristics was used. This POF has a core diameter of 0.98 mm and an outer diameter of 1 mm. The core material is PMMA resin and the cladding is made of fluorinated polymer. The POF is covered by a black jacket based on polyethylene to shield extraneous light. The refractive indices of the core and the cladding are 1.49 and 1.402, respectively; therefore, the NA is approximately 0.50. The POF length is about 6 m, which is a sufficient length considering the distance between the examination room and the control room.

**Figure 1:**
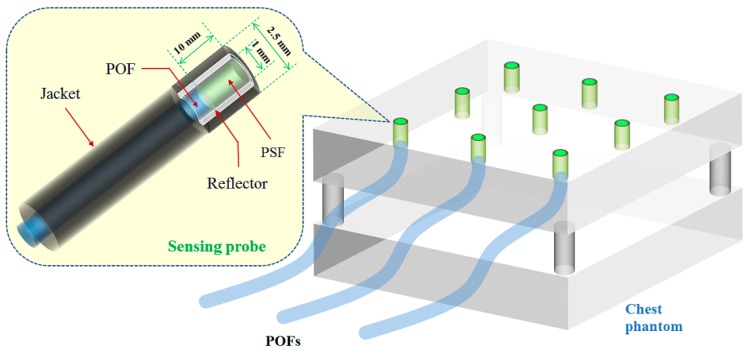
Inner structure of the sensing probe and the arrangement of nine sensing probes at each center of nine equal parts on the top of the ANSI sensitometry chest phantom.

The inner structure of the sensing probe is described in Figure 1. A 10 mm long PSF was polished and coupled to a 6 m long POF. The PSF was then covered by a reflective paint (BC-620, Saint-Gobain Ceramic & Plastics, Hiram, OH, USA) based on titanium dioxide (TiO_2_) to improve the light collection efficiency. In addition, the sensing probe was wrapped with black tape to shield ambient light. Before the experiments, we first selected nine promising probes which have almost identical light output by using a multi-pixel photon counter (MPPC: S10362-11-100U, Hamamatsu Photonics, Hamamatsu, Japan). We also obtained the calibration factor by measuring the light intensities of all probes at the same position. 

As a chest phantom, an American National Standards Institute (ANSI) sensitometry chest phantom was fabricated [3]. The description and use of the ANSI sensitometry chest phantom is proposed in report No. 31 which is published for the American Association of Physicists in Medicine (AAPM) by the American Institute of Physics (AIP) [13]. As can be seen in Figure 1, the top of the chest phantom was divided into nine equal parts virtually and we made nine holes with a diameter of 2.5 mm at each center of the nine equal parts on the top of the chest phantom. The distal ends of nine sensing probes with an identical structure were installed at each center of the nine equal parts as measuring points (*i.e.*, channel 1–9) to measure the planar dose distribution. The transmitting POFs of nine sensing probes were taken in the form of a coherent bundle with a 3 × 3 square array by using a plastic holder and then connected to an image intensifier (MCP-PROXIFIER^®^, ProxiVision GmbH, Bensheim, Germany) with a fiber-optic window and a useful diameter of 40 mm to increase the light intensity of the scintillation signals transmitted via POFs.

### 2.2. Multichannel All-In-One Phantom Dosimeter System for Measuring Planar Dose Distribution of X-ray Beam

**Figure 2:**
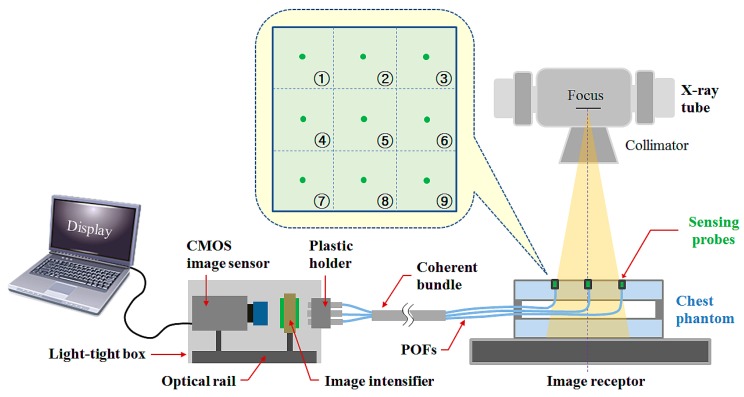
Experimental setup using a multichannel all-in-one phantom dosimeter and a DR system.

Figure 2 illustrates the experimental setup to obtain the planar dose distribution on a chest phantom using the multichannel all-in-one phantom dosimeter system. The performance evaluation of the proposed phantom dosimeter was carried out using an X-ray tube (Rotanode^TM^ E7252X, Toshiba Electron Tubes & Devices, Saitama, Japan) of a digital radiography (DR) system (Clear Vision DR 7000F, JPI Healthcare, Osong, Korea) with 0.6/1.2 mm dual focal spots, a 12° rhenium-tungsten-faced molybdenum target, and 0.9 mm Al filtration at 75 kVp [7]. When the sensing probes installed at the top of the chest phantom are irradiated by an X-ray beam, the scintillation signals are transmitted through the POFs and their light intensities are intensified by the image intensifier. To intensify the scintillation signals, it performs the following three main processes. First, a bialkali photocathode with a composition of K_2_SbCs on the distal end of an image intensifier converts the entered photons into electrons. The electrons are then accelerated through the microchannel plates (MCPs). Finally, the phosphor screen with a composition of Gd_2_O_2_S:Tb on the proximal end of the image intensifier converts electrons back into photons. 

The real-time scintillation images (RSIs) including the intensified nine scintillation signals are taken by a CMOS image sensor (acA3800-14uc, BASLER, Ahrensburg, Germany) with a single lens optical system. The RSIs are converted into red-green-blue (RGB) image files by the MATLAB program (MATLAB^®^, MathWorks, Natick, MA, USA) and subsequently transformed to gray scale. The gray-scale image format is corrected by multiplying the calibration factor acquired from a reference parameter.

Using nine sensing probes of the phantom dosimeter system that are installed in the upper part of the chest phantom, the RSIs were measured to obtain the planar dose distribution, which changed with the variation of the X-ray exposure parameters. We then compared the results with the absorbed doses measured by using a conventional SCD (ALPHA plus, Pehamed, Sulzbach, Germany) with a finger-shaped dose sensor. In this study, the values of the exposure parameters such as the beam field size, focus-to-image receptor distance (FID), tube potential, and current-time product were fixed at 30 × 30 cm^2^, 100 cm, 100 kVp, and 5 mAs, respectively.

## 3. Experimental Results

### 3.1. Measurement of Planar Dose Distribution

**Figure 3:**
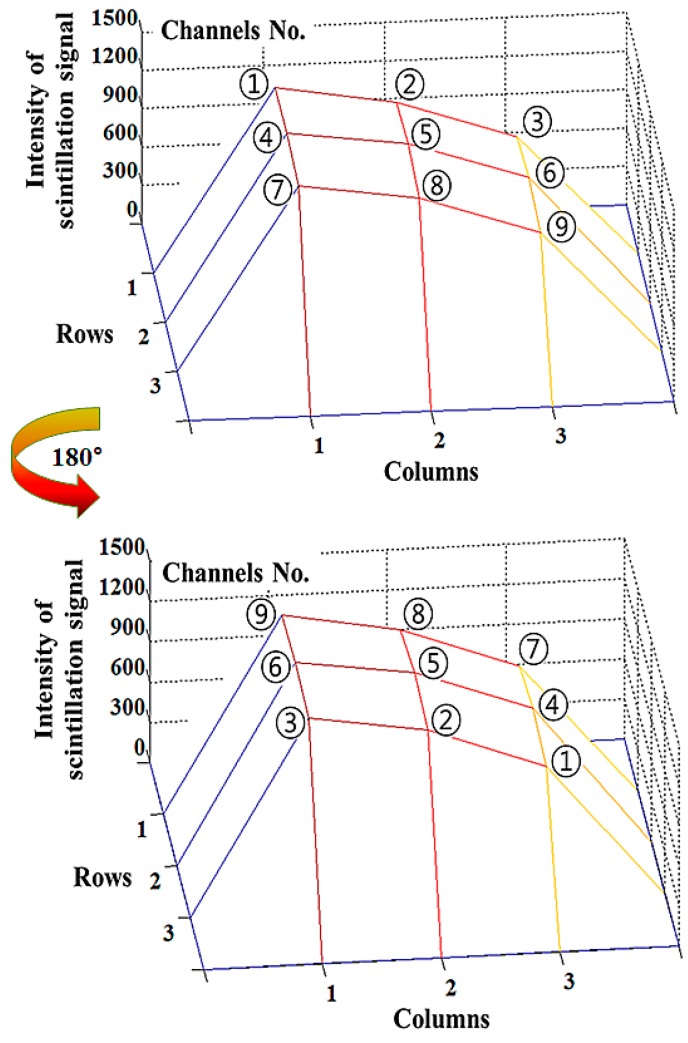
Intensity distribution of the scintillation signals measured by using the multichannel all-in-one phantom dosimeter when the phantom dosimeter was placed at 0° and 180° during beam irradiation with a tube potential of 150 kVp.

To evaluate the performance of the multichannel all-in-one phantom dosimeter, we first measured the planar dose distribution during X-ray beam irradiation. Generally, the intensity distribution of the X-ray beam (*i.e.*, beam profile) is influenced by the target angle of the X-ray tube and it is commonly called the heel effect. To verify whether dose non-uniformity is mostly affected by the heel effect, nine scintillation signals were measured simultaneously by using the phantom dosimeter. As expected, the intensity distribution of the scintillation signals was not uniform at each measuring point (*i.e.*, channel 1–9 as shown in Figure 2) on the chest phantom, as shown in Figure 3. Furthermore, we measured the intensity distribution of scintillation signals in the RSI when the phantom dosimeter was placed at 0° and 180°. As can be seen in Figure 3, although the position of the phantom dosimeter is changed, the intensity distribution of the scintillation signals affected by the heel effect is measured normally regardless of the sensing probes, and it was equal to the planar dose distribution on the chest phantom obtained by using the SCD. Therefore, we demonstrated that the planar dose distribution can be easily and quickly measured by using the multichannel all-in-one phantom dosimeter system. 

At channel No. 5 (Ch. 5) on the center of the entrance surface in the X-ray beam field, we also obtained the intensity of the scintillation signal relating the ESD value to evaluate the patient dose in diagnostic radiology. If a modified direct dose measurement is applied to the multichannel all-in-one phantom dosimeter, the ESD value can be inferred easily by using the intensities of the scintillation signals measured at the edges (*i.e.*, all channels except Ch. 5) on the chest phantom [8].

### 3.2. Performance Evaluation of a Multichannel All-In-One Phantom Dosimeter According to the Tube Potential

**Figure 4:**
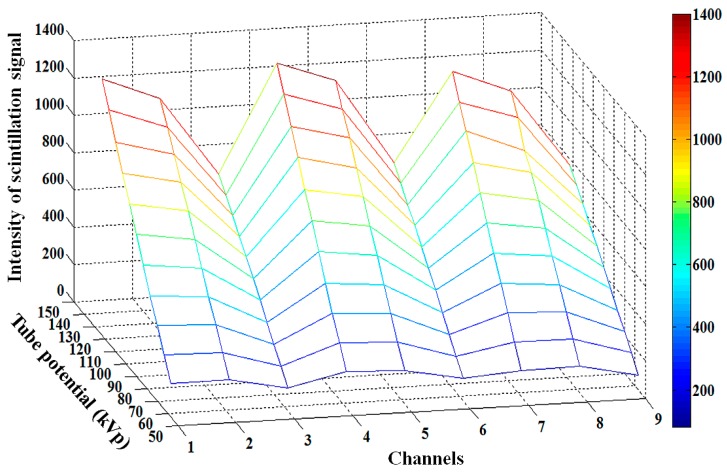
3D intensity variation of nine scintillation signals measured by using the proposed phantom dosimeter at each measuring point in accordance with the tube potential.

Next, we carried out the experimental study on the performance evaluation of a multichannel all-in-one phantom dosimeter according to the tube potential. Figure 4 shows the three-dimensional (3D) intensity variation of nine scintillation signals measured by using the proposed phantom dosimeter at each measuring point according to the variation of the tube potential with 10 kVp intervals. The intensity of the scintillation signal at each measuring point was obtained by summating the gray-scale values within the regions of interest (ROI) in the RSI using the MATLAB program. As the tube potential increased, all intensities of the scintillation signals also increased.

**Figure 5:**
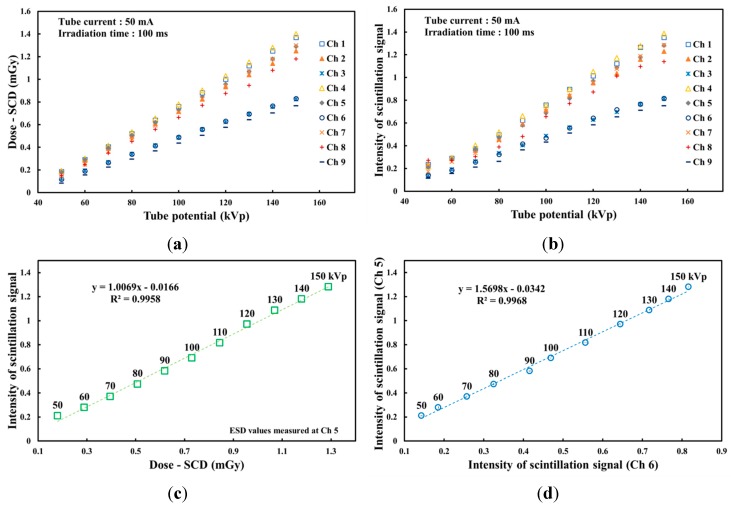
Variations of (**a**) the absorbed dose of the SCD and (**b**) the light intensity of the phantom dosimeter according to the tube potential. (**c**) Relationship between the light intensities of the phantom dosimeter and the ESDs of the SCD at Ch. 5 and (**d**) the correlation between two probes of the phantom dosimeter at each measuring point as a function of the tube potential.

By using the SCD and the multichannel all-in-one phantom dosimeter, the absorbed dose and the RSI were measured, respectively, in accordance with the tube potential, as shown in Figure 5. As the tube potential was increased, the absorbed dose of the SCD and the light intensity of the phantom dosimeter increased over the X-ray tube potential range of interest in diagnostic radiology. In this test, the absorbed doses were measured sequentially by using the SCD with a finger-shaped dose sensor at each measuring point. On the other hand, the intensities of the nine scintillation signals in the RSI were measured simultaneously by using the phantom dosimeter at all measuring points. In the results, it is found that all absorbed doses and light intensities at nine measuring points have increasing trends with different gradients in the tube potential range from 50 kVp to 150 kVp, as shown in Figure 5a,b. 

Based on the experimental results respecting the light intensities of the scintillation signals measured at nine measuring points, probe 6, which is installed at Ch. 6, among the nine sensing probes was selected as a promising probe to infer the ESD values measured at Ch. 5. Figure 5c plots the relationship between the light intensities of the phantom dosimeter and the ESDs of the SCD on the center of the entrance surface according to the tube potential. According to the increase of the tube voltage, both the light intensities and ESDs increased during X-ray irradiation with diagnostic energies. By using the relationship between the light intensity of the phantom dosimeter and the absorbed dose of the SCD at each channel, it is possible to convert the intensity values of the scintillation signal to the absorbed dose values. Figure 5d shows the correlation between the two probes of the phantom dosimeter, which are installed at Chs. 5 and 6, respectively, as a function of the tube potential. Using the correlation in Figure 5c,d with the modified direct dose measurement, the ESD values can be inferred easily by the scintillating light intensities measured at Ch. 6 [8].

### 3.3. Performance Evaluation of a Multichannel All-In-One Phantom Dosimeter as a Function of the Current-Time Product

**Figure 6:**
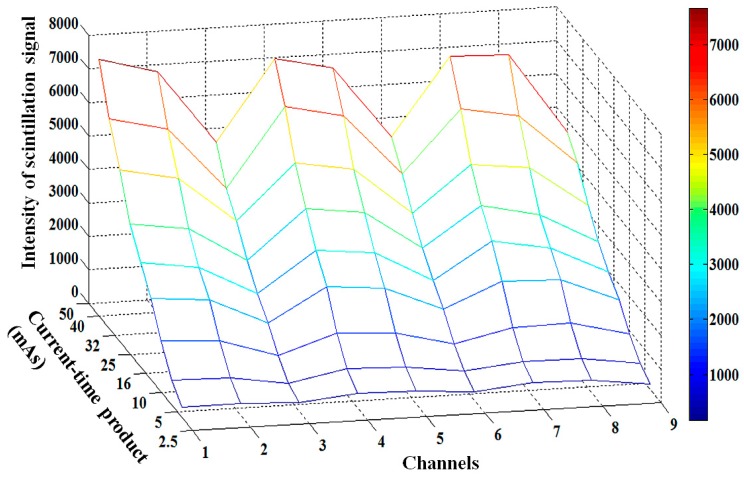
3D intensity variation of nine scintillation signals measured by using the proposed phantom dosimeter at each measuring point according to the current-time product.

Finally, the performance of the multichannel all-in-one phantom dosimeter was also evaluated by changing the tube current from 25 mA to 500 mA while keeping the exposure time constant at 100 ms. Therefore, the current-time product was changed from 2.5 mAs to 50 mAs. Figure 6 shows the 3D intensity variation of the nine scintillation signals measured by using the proposed phantom dosimeter at each measuring point according to the variation of the current-time product. As the current-time product increased, all intensities of the scintillation signals at nine measuring points also increased.

**Figure 7:**
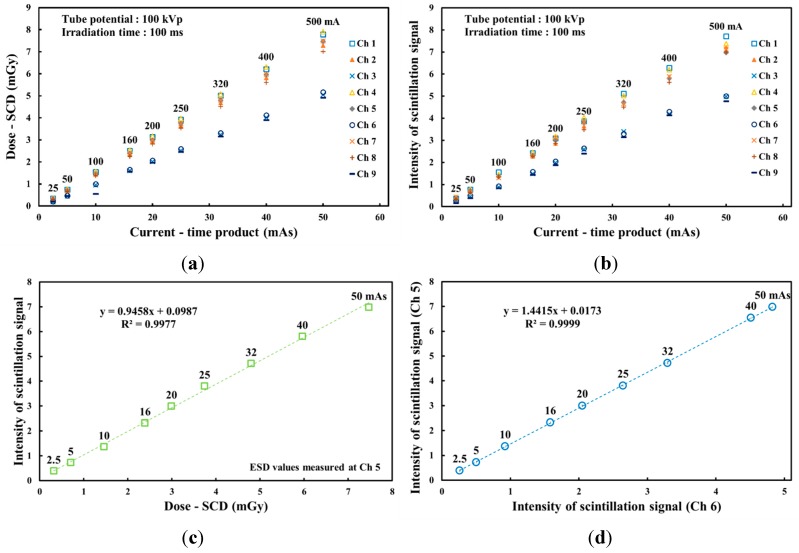
(**a**) Absorbed doses measured using the SCD and (**b**) light intensity measured using the phantom dosimeter from each probe. (**c**) Relationship between the light intensities of the phantom dosimeter and the ESDs of the SCD at Ch. 5 according to the current-time product. (**d**) Correlation between two probes of the phantom dosimeter at each measuring point according to the current-time product.

Figure 7 shows the absorbed dose and light intensity measured by the SCD and the phantom dosimeter, respectively, with variation of the current-time product. As can be seen in Figure 7a, all the absorbed doses at nine measuring points also increased according to the increase of the tube current, similar to the result in Figure 5a. The gradients of the absorbed doses measured at Chs. 1, 4 and 7 are steeper than those of absorbed doses at Chs. 3, 6 and 9 according to the variation of the tube voltage and current, as shown in Figure 3, Figure 5a and Figure 7a. Figure 7c shows the relationship between the light intensities of the phantom dosimeter and the ESDs of the SCD measured at the center of the irradiation field as a function of the current-time product. Both the light intensity of the phantom dosimeter and the absorbed dose of the SCD linearly increased as the current-time product increased from 2.5 to 50 mAs. From the results of Figure 5c and Figure 7c, it can be realized that the proposed phantom dosimeter has a linear response according to the dose variation of the X-ray beam. Additionally, the intensity of the scintillation signals can be calculated to the absorbed dose values by using the relationship between the obtained data, similar to the result in Figure 5c. Figure 7d shows the correlation between the two probes of the phantom dosimeter at Chs. 5 and 6 in accordance with the current-time product. The light intensities measured at Ch. 5 are higher than those of Ch. 6 and the R^2^ value of the linear line was found to be 0.9999.

## 4. Discussion and Conclusions

In this study, we encouraged the apprehension of over-exposure due to the heel effect in part of the patient's body during chest radiography. To obtain an X-ray beam profile, we measured the light intensities of scintillation signals due to the X-ray beam and demonstrated that the planar dose distribution including the ESD can be easily and quickly measured by using the proposed multichannel all-in-one phantom dosimeter system. From the experimental results, we confirmed that the beam profile of an X-ray used in diagnostic radiology is affected predominantly by the heel effect and the absorbed dose which is higher than the ESD values can be delivered to some other points, not the center of the beam irradiation field. 

In conclusion, it is expected that the proposed phantom dosimeter will be a useful dosimeter to measure the real-time dose distribution and the beam quality of a DR system and to select the adequate measuring point for application of the modified direct measurement before chest radiography. Further studies will be conducted to develop a newly designed multichannel all-in-one phantom dosimeter system to accurately measure the planar and depth dose distribution with enhanced spatial resolution.

## References

[1] Andria G., Nisio A.D., Lanzolla A.M.L., Guglielmi G., Terlizzi R. (2014). Dose Optimization in Chest Radiography: System and Model Characterization via Experimental Investigation. IEEE Trans. Instrum. Meas..

[2] Gislason-Lee A.J., McMillan C., Cowen A.R., Davies A.G. (2013). Dose optimization in cardiac X-ray imaging. Med. Phys..

[3] Yoo W.J., Jeon D.J., Seo J.K., Shin S.H., Han K.-T., Youn W.S., Cho S., Lee B. (2013). Development of a scintillating fiber-optic dosimeter for measuring the entrance surface dose in diagnostic radiology. Radiat. Meas..

[4] Soldt R.T.M.V., Zweers D., Berge L.V.D., Geleijns J., Jansen J.T.M., Zoetelef J. (2014). Survey of posteroanterior chest radiography in the Netherlands: Patient dose and image quality. Brit. J. Radiol..

[5] Tsapaki V., Tsalafoutas I.A., Chinofoti I., Karageorgi A., Carinou E., Kamenopoulou V., Yakoumakis E.N., Koulentianos E.D. (2007). Radiation doses to patients undergoing standard radiographic examinations: A comparison between two methods. Brit. J. Radiol..

[6] Conway B.J., Butler P.F., Duff J.E., Fewell T.R., Gross R.E., Jennings R.J., Koustenis G.H., McCrohan J.L., Rueter F.G., Showalter C.K. (1984). Beam quality independent attenuation phantom for estimating patient exposure from x-ray automatic exposure controlled chest examinations. Med. Phys..

[7] Yoo W.J., Shin S.H., Jeon D.J., Hong S., Sim H.I., Kim S.G., Jang K.W., Cho S., Youn W.S., Lee B. (2014). Measurement of entrance surface dose on an anthropomorphic thorax phantom using a miniature fiber-optic dosimeter. Sensors.

[8] Yoo W.J., Jeon D.J., Seo J.K., Shin S.H., Han K-T., Hong S., Kim S.G., Cho S., Lee B. (2013). Development of a fiber-optic dosimeter based on modified direct measurement for real-time dosimetry during radiation diagnosis. Meas. Sci. Technol..

[9] Hyer D.E., Fisher R.F., Hintenlang D.E. (2009). Characterization of a water-equivalent fiber-optic coupled dosimeter for use in diagnostic radiology. Med. Phys..

[10] Jones A.K., Hintenlang D. (2008). Potential clinical utility of a fibre optic-coupled dosemeter for dose measurements in diagnostic radiology. Rad. Prot. Dosim..

[11] Benevides L.A., Huston A.L., Justus B.L., Falkenstein P., Brateman L.F., Hintenlang D.E. (2007). Characterization of a fiber-optic-coupled radioluminescent detector for application in the mammography energy range. Med. Phys..

[12] Ardran G.M., Crooks H.E. (2014). Checking diagnostic X-ray beam quality. Brit. J. Radiol..

[13] Chu R.Y.L., Fisher J., Archer B.R., Conway B.J., Goodsitt M.M., Glaze S., Gray J.E., Strauss K.J. (1990). Standardized Methods for Measuring Diagnostic X-ray Exposures.

